# Establishment and function of tissue-resident innate lymphoid cells in the skin

**DOI:** 10.1007/s13238-017-0388-4

**Published:** 2017-03-07

**Authors:** Jie Yang, Luming Zhao, Ming Xu, Na Xiong

**Affiliations:** 1 Centre for Molecular Immunology and Infectious Diseases and Department of Veterinary and Biomedical Sciences, The Pennsylvania State University, University Park, PA 16802 USA; 2 Department of Immunology, The University of Texas MD Anderson Cancer Center, 7455 Fannin Street, Houston, TX 77054 USA

**Keywords:** innate lymphoid cells, skin, migration, chemokine receptor, homeostasis, inflammation

## Abstract

Innate lymphoid cells (ILCs) are a newly classified family of immune cells of the lymphoid lineage. While they could be found in both lymphoid organs and non-lymphoid tissues, ILCs are preferentially enriched in barrier tissues such as the skin, intestine, and lung where they could play important roles in maintenance of tissue integrity and function and protection against assaults of foreign agents. On the other hand, dysregulated activation of ILCs could contribute to tissue inflammatory diseases. In spite of recent progress towards understanding roles of ILCs in the health and disease, mechanisms regulating specific establishment, activation, and function of ILCs in barrier tissues are still poorly understood. We herein review the up-to-date understanding of tissue-specific relevance of ILCs. Particularly we will focus on resident ILCs of the skin, the outmost barrier tissue critical in protection against various foreign hazardous agents and maintenance of thermal and water balance. In addition, we will discuss remaining outstanding questions yet to be addressed.

## INTRODUCTION

Innate lymphoid cells (ILCs) are the newly identified members of the innate immune system. ILCs have the property of classic lymphoid cells but lack rearranged antigen-specific receptors (Spits et al., 2013; Walker et al., 2013). ILCs encompass conventional natural killer (NK) cells and lymphoid tissue inducer (LTi) cells, which were first discovered in 1975 and 1997, respectively (Kiessling et al., 1975a; Kiessling et al., 1975b; Mebius et al., 1997), and other recently described subsets. ILCs can be generally identified by their expression of certain subunits of cytokine receptors such as CD25 (interleukin (IL)-2 receptor α) and CD127 (IL-7 receptor α) but no cell-surface molecules that identify the other types of immune cells (lineage marker-negative or Lin^−^) (Artis and Spits, 2015). While ILCs could be found in nearly every tissue and organ, they are preferentially enriched in barrier tissues such as the skin, intestine and lung, where they participate in local tissue homeostasis and inflammation (Artis and Spits, 2015; McKenzie et al., 2014).

## SUBSETS OF INNATE LYMPHOID CELLS

In analog to helper T (Th) cells, ILCs are conventionally divided into three groups based on their differential expression of effector cytokines and developmental requirements for transcription factors (Spits et al., 2013). The group 1 ILCs are composed of ILCs capable of producing interferon γ (IFNγ), which include NK cells and non-cytotoxic helper-like ILC1s. NK cells are innate effector lymphocytes that are capable of inducing granule-mediated cytotoxicity by expressing perforin and granzymes, as well as producing IFNγ and tumor necrosis factor α (TNFα) (Herberman et al., 1975a; Herberman et al., 1975b; Kiessling et al., 1975a; Kiessling et al., 1975b; Vivier et al., 2011). On the other hand, non-cytotoxic ILC1s are innate helper lymphocytes that are capable of producing IFNγ, TNFα and other Th1-associated cytokines, but lack cytotoxic abilities (Artis and Spits, 2015; Bernink et al., 2013; Vonarbourg et al., 2010). Recent studies identified additional developmental and phenotypic differences between NK and ILC1s (Constantinides et al., 2014; Klose et al., 2014). While eomesodermin (Eomes) is required for development of NK cells, T-bet and GATA3 are critical for ILC1s (Daussy et al., 2014; Gordon et al., 2012; Yagi et al., 2014). In addition, NK cells re-circulate in the blood while ILC1s predominantly reside in tissues (Sojka et al., 2014). Group 2 ILCs produce Th2-associated cytokines such as IL-4, IL-5, and IL-13 when activated, and depend on GATA3 and RORα for their development (Halim et al., 2012; Hoyler et al., 2012). Group 3 ILCs are highly heterogeneous, although they preferentially produce Th17-assciated cytokines including IL-17A and IL-22 and require RORγt for the development. Based on their expression of the chemokine receptor CCR6, group 3 ILCs can be divided into two subsets (Klose et al., 2013; Sawa et al., 2011). CCR6^+^ ILC3s consist of CD4^+^ and CD4^−^ LTi cells, while CCR6^−^ ILC3s are composed of two subpopulations that have distinct expression of the natural cytotoxicity receptor (NCR) NKp46 (Klose et al., 2013; Rankin et al., 2013).

ILCs of the different groups can show certain functional plasticity. For example, in response to IL-12 and IL-18, CCR6^−^NKp46^−^ ILC3s increase expression of T-bet and differentiate into CCR6^−^NKp46^+^ ILC3s, which can further increase T-bet and decrease RORγt expression and differentiate into ex-RORγt^+^ ILC3s that display ILC1-like functions with increased production of IFNγ and decreased IL-17A and IL-22 (Artis and Spits, 2015; Bernink et al., 2013; Cella et al., 2010; Klose et al., 2013; Vonarbourg et al., 2010). Using the single cell transcription analysis, a recent study found that there are significant heterogeneities within ILCs and that some of them display transcription profiles associated with more than one group of ILCs, consistent with the notion that they are of transition stages (Gury-BenAri et al., 2016).

## DEVELOPMENT OF INNATE LYMPHOID CELLS

Common lymphoid progenitor cells of the bone marrow give rise to all lymphocytes, including ILCs. Several intermediate progenitors have been identified that have the potential to give rise to different subsets of ILCs. Committed ILC precursors can differentiate into all ILC subsets, including NK cells, but not T and B cells (Seillet et al., 2014; Yu et al., 2014). They are termed α-lymphoid precursor (αLP) cells because they express the integrin α4β7, but also known as common innate lymphoid progenitor (CILP). αLP cells express the chemokine receptor CXCR6 and require the transcription factor NFIL3 for development (Seillet et al., 2014; Yu et al., 2014). Downstream of αLP, two populations of ILC precursor cells have been identified based on the expression of inhibitor of DNA binding 2 (Id2) and promyeloid leukemia zinc finger (PLZF) (Constantinides et al., 2014; Klose et al., 2014). Common helper-like innate lymphoid progenitor (CHILP) cells are Id2^+^Lin^−^IL-7Rα^+^α4β7^+^CD25^−^ and dependent on GATA3 for development. CHILP can give rise to non-cytotoxic ILC1s, ILC2s and ILC3s, but no NK cells (Klose et al., 2014). Innate lymphoid progenitor (ILCP) cells are PLZF^high^Lin^−^IL-7Rα^+^cKit^+^α4β7^high^CXCR6^−^. ILCP differentiates efficiently into the majority of ILC subsets except NK cells and LTi cells (Constantinides et al., 2014). ILCP is thus placed downstream of CHILP because ILCP has a more restricted differentiation potential than CHILP. More recently, a population of the transcription factor TCF-1^+^ early ILC progenitor (EILP) cells were also described (Yang et al., 2015). The TCF-1^+^ EILP cells are IL-7Rα^neg/low^Thy1^−^Lin^−^CD122^−^CXCR6^−^ and express low levels of PLZF. EILP could develop into all subsets of ILCs, including conventional NK cells, but not T/B cells (Yang et al., 2015). TCF-1 knockout mice have reduced EILP as well as NKP and CHILP, suggesting that TCF-1 is critical for development of EILP and that NKP and CHILP are downstream of EILP (Yang et al., 2015). The relationship between EILP and αLP is not fully characterized even though EILP seems to functionally overlap with αLP in their ability to differentiate into ILCs but not T/B cells. Detailed developmental pathway and regulation of the ILC differentiation have been extensively reviewed (Ishizuka et al., 2016; Serafini et al., 2015; Yang and Bhandoola, 2016; Zook and Kee, 2016).

## FUNCTION OF INNATE LYMPHOID CELLS IN MAINTENANCE OF TISSUE HOMEOSTASIS AND HOST PROTECTION

ILCs are enriched in barrier tissues where they play important roles in maintenance of tissue homeostasis, regulation of equilibrium between the host and microbiota, and immunity against pathogens (Artis and Spits, 2015; McKenzie et al., 2014; Walker et al., 2013). In addition, ILCs participate in the development of lymphoid tissues, tissue remodeling, and metabolic homeostasis (Artis and Spits, 2015; McKenzie et al., 2014; Walker et al., 2013).

Functions of NK cells of the group 1 ILC family have been well established, which include killing virus-infected cells through cytolysis mediated by granzymes and perforin, and targeting tumor cells that have lost expression of the class I major histocompatibility complex (MHCI) (Biron et al., 1999; Vivier et al., 2012). The other non-cytotoxic ILC1s can produce high amounts of IFNγ and TNFα, contributing to the protection of hosts against bacteria and intracellular parasites (Klose et al., 2014; Klose et al., 2013). More recently, it was also reported that ILC1s play an important role in the early protection against tumor genesis (Dadi et al., 2016).

ILC2s are a critical innate source of type 2 cytokines, such as IL-4, IL-5, IL-9 and IL-13, which can induce eosinophilia, mastocytosis, activation of alternatively activated macrophages, goblet cell hyperplasia and muscle contractility, and tissue repair (Pulendran and Artis, 2012). ILC2s can be activated by cytokines such as IL-25, IL-33, and thymic stromal lymphopoietin (TSLP) (Hams et al., 2014; Kim et al., 2013; McHedlidze et al., 2013). During parasitic helminth infection, ILC2s are identified as an important source of IL-13 critical for the parasite expulsion (Moro et al., 2016; Neill et al., 2010; Price et al., 2010). ILC2s also express amphiregulin and are involved in tissue repairing after virus infection (Monticelli et al., 2011). In addition, growing evidence demonstrates roles of ILC2s in metabolic homeostasis (Hams et al., 2013; Molofsky et al., 2013; Stanya et al., 2013). ILC2s in visceral adipose tissue produce IL-5 and IL-13 and maintain responses of eosinophil and alternatively activated macrophages, lack of which results in increased adiposity and insulin resistance (Molofsky et al., 2013). ILCs can respond to changes in nutrients such as Vitamin A, deficiency of which leads to reduced ILC3s but increased ILC2s in intestines and is associated with defective antibacterial immunity but enhanced anti-helminth immunity (Spencer et al., 2014). Circadian clock also regulates cytokine production of intestinal ILC2s through vasoactive intestinal peptides (Nussbaum et al., 2013).

The role of LTi cells of the group 3 ILCs family in the development of lymphoid organs of lymph nodes (LNs) and Peyer’s patches has long been recognized (De Togni et al., 1994; Eberl et al., 2004). Lymphotoxin α1β2 produced by LTi cells binds to lymphotoxin β receptor on stromal cells to induce the production of chemokines and upregulation of adhesion molecules, which recruit leukocytes to form lymphoid structures (van de Pavert et al., 2009). ILC3s have also been implicated in tissue repair following inflammation or damage in multiple tissues, including intestines, thymi, and lungs (Dudakov et al., 2012; Sawa et al., 2011; Taube et al., 2011). ILC3s are an important source of IL-22, which promotes epithelial cells to express antimicrobial peptides to protect against pathogens (Sonnenberg et al., 2011; Zheng et al., 2008). In addition, ILC3s interact with stromal cells and other immune cells to maintain balance between the host and microbiota by limiting dissemination of commensal bacteria and inappropriate immune responses to them (Sonnenberg et al., 2012).

## PARTICIPATION OF INNATE LYMPHOID CELLS IN PATHOGENESIS OF DISEASES

Although ILCs normally play host-protective roles, dysregulated activation of ILCs could lead to inflammatory diseases (Artis and Spits, 2015; McKenzie et al., 2014; Walker et al., 2013). IFNγ-producing ILCs, including ILC1s and ex-RORγt^+^ ILC3s, can induce inflammation in the intestines of both mice and humans (Bernink et al., 2013; Buonocore et al., 2010; Fuchs et al., 2013). In the adipose tissue, high fat diet could activate ILC1s to produce IFNγ and promote inflammatory macrophage polarization and development of insulin resistance (O’Sullivan et al., 2016).

Chronic ILC2 activation was reported to contribute to a large variety of tissue inflammatory disorders such as asthma in the lung and atopic dermatitis in the skin, which are generally associated with over-production of the type 2 inflammatory cytokines such as IL-4, IL-5, and IL-13. ILC2s expand and produce the type 2 cytokines in mice with allergic lung inflammation (Chang et al., 2011; Halim et al., 2014), consistent with the increase of ILC2s in peripheral blood of patients with asthma (Bartemes et al., 2014; Moffatt et al., 2010). Increased frequency of ILC2s was found in lesional skin of mouse models and human patients with atopic dermatitis (Imai et al., 2013; Kim et al., 2013; Salimi et al., 2013).

ILC3s could contribute to inflammatory diseases and tumorigenesis in various tissues. IL-17- and IL-22-producing ILC3s have been linked with the inflammatory skin disease psoriasis, inflammatory bowl disease, obesity-associated asthma, and multiple sclerosis (Buonocore et al., 2010; Kim et al., 2014b; Pantelyushin et al., 2012; Perry et al., 2012; Powell et al., 2012; Teunissen et al., 2014; Villanova et al., 2014). IL-22 produced by ILC3s can contribute to development of colon cancer in a mouse model, consistent with the presence of IL-22-producing CD3^−^ cells within human colorectal carcinoma tumors (Kirchberger et al., 2013).

## INNATE LYMPHOID CELLS ARE PREDOMINANTLY TISSUE-RESIDENT CELLS

Specific functions of ILCs in homeostasis and inflammation are associated with their unique localization in peripheral tissues. Recently, several groups reported that ILCs are predominantly tissue-resident cells that do not circulate in the body. In parabiotic studies, helper-like ILCs in both non-lymphoid tissues and lymphoid organs such as the small intestine, salivary gland, lung, mensenteric lymph node, and adipose tissue of two joined adult mice with shared blood circulation systems remain locally and do not move from one mouse to another (Gasteiger et al., 2015; O’Sullivan et al., 2016). However, conventional NK cells are mostly circulating cells (Peng et al., 2013; Sojka et al., 2014). Furthermore, systemic inflammation or infection did not mobilize tissue-resident ILCs into the circulation either (Gasteiger et al., 2015). These suggest that ILCs or their progenitor cells might settle in specific peripheral tissues at early ontogenic stages and are maintained and expanded locally. Consistent with this notion, progenitor cells of ILCs have been identified in lymph nodes of humans that could give rise to all innate lymphoid cells in the *in vitro* development analysis (Scoville et al., 2016). In addition, the bone marrow reconstitution in human patients with severe combined immunodeficiency (SCID) due to mutation of the gene encoding the common γ chain cytokine receptor subunit IL-2Rγ or the tyrosine kinase JAK3 did not restore presence of ILCs in various peripheral tissues (Vely et al., 2016), supporting the notion that tissue-resident ILCs are established at early ontogenic stages. Mechanisms regulating specific localization and maintenance of ILCs in various peripheral tissues are still poorly understood.

## MIGRATION AND ESTABLISHMENT OF INNATE LYMPHOID CELLS IN THE SKIN

Skin is the outmost barrier tissue constantly exposed to assaults of various foreign agents. Skin is enriched with ILCs and ILCs of all the three groups could be found in the skin (Yang et al., 2016). Based on expression of the transcription factor GATA3 and the cytokines such as IL-4 and IL-5, ILC2s account for a major fraction of total skin ILCs in adult mice (Roediger et al., 2013; Yang et al., 2016). Although few ILCs of the skin in mice express the transcription factor RORγt, a significant fraction of skin ILCs are capable of producing IL-17A, suggesting that they are ILC3s or ILC3-like cells independent of RORγt (Yang et al., 2016). However, their lineage relationship with other RORγt-expressing ILC3s is not known. The group 1 ILCs, including NK cells, are present in the skin of mice (Luci et al., 2009; Yang et al., 2016), and they account for a smaller fraction of total skin ILCs than ILC2s or ILC3s in adult mice (Yang et al., 2016). On the other hand, in the skin of fetal and newborn mice, there are abundant NK1.1^+^ ILC1-type cells although their origin and lineage association are not known (Almeida et al., 2015). As in mice, ILCs of all the three groups are found in the healthy skin of humans (Dyring-Andersen et al., 2014; Ebert et al., 2006; Salimi et al., 2013; Teunissen et al., 2014; Villanova et al., 2014). Based on their expression of the prostaglandin D2 receptor CRTH2, ILC2s account for 25%–40% of skin ILCs in humans (Dyring-Andersen et al., 2014; Salimi et al., 2013; Teunissen et al., 2014; Villanova et al., 2014). ILC3s, including both NCR^+^ and NCR^−^ subsets, account for about 50% of total skin ILCs in humans (Dyring-Andersen et al., 2014; Teunissen et al., 2014; Villanova et al., 2014). The rest are ILC1s and others uncharacterized.

**Figure 1:**
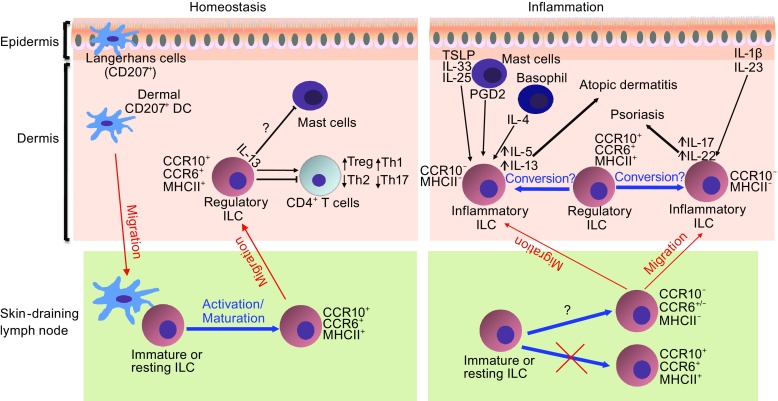
**Schematic illustration of establishment and function of ILCs in the skin homeostasis and inflammation.** Under homeostatic conditions, CCR10^+^ ILCs with regulatory properties are preferentially generated in skin-draining lymph nodes and migrate into the skin where they promote the local immune homeostasis by controlling balanced presence and activation of other immune cells. Under inflammatory conditions, there is reduced generation of CCR10^+^ regulatory ILCs in skin-draining lymph nodes. In addition, there is also increased conversion of regulatory CCR10^+^ ILCs to inflammatory CCR10^−^ ILCs, which promote immune activation and inflammatory processes in diseases such as atopic dermatitis and psoriasis, depending cytokines produced by the activated inflammatory ILCs

Since ILCs share many common regulatory pathways with helper T cells for their development and function, we investigated whether skin-specific ILCs are programmed in skin-draining lymph nodes to acquire their skin-homing property for establishment of their skin residency, paralleling the process by which the skin-homing property of conventional αβ T cells is imprinted in skin-draining lymph nodes (Yang et al., 2016). Among the most skin-specific homing molecules expressed on skin-homing T cells and ILCs is the chemokine receptor CCR10 (Sigmundsdottir and Butcher, 2008; Sigmundsdottir et al., 2007; Xiong et al., 2012; Yang et al., 2016). A ligand for CCR10, CCL27, is highly and specifically expressed by keratinocytes of the healthy skin in both humans and mice (Homey et al., 2000; Morales et al., 1999). The adhesion molecules E- and P-selectin ligands and chemokine receptors CCR4, CCR6, and CCR8 are also involved in the skin-homing process in the healthy skin (Austrup et al., 1997; Campbell and Butcher, 2002; Campbell et al., 1999; Picker et al., 1993; Reiss et al., 2001; Weninger et al., 2000). In addition, different sets of homing molecules are involved in migration of immune cells to the inflamed skin under specific inflammatory conditions than those under homeostatic conditions (Lonsdorf et al., 2009; Masopust and Schenkel, 2013; Mora and von Andrian, 2006). For example, high induction of the chemokines CCL1 and CCL18 is found in the lesional skin of patients with atopic dermatitis (Gombert et al., 2005; Pivarcsi et al., 2004). The chemokine receptor CXCR3 is involved in migration of T cells to the inflamed skin, where its ligands CXCL9, CXCL10, and CXCL11 are produced (Flier et al., 2001). While CCL27 could be upregulated in the skin under inflammatory conditions of allergic contact dermatitis, atopic dermatitis, and psoriasis (Homey et al., 2002; Moed et al., 2004), it is profoundly suppressed in severe psoriatic skin lesions (Gudjonsson et al., 2010; Quaranta et al., 2014; Riis et al., 2011a). In the same patient, CCL27 expression is much lower in psoriatic lesional skin than in peri-lesional skin (Karakawa et al., 2014; Riis et al., 2011a), suggesting that its expression is associated with severity of the disease. Indeed, CCL27 is upregulated in the skin by the inflammatory cytokines TNFα and IL-1β at the early phase of inflammation (Riis et al., 2011b; Vestergaard et al., 2004) but is suppressed by pathognomonic cytokines such as IL-17A and IFNγ at the late, severe stage of skin inflammation (Kanda et al., 2005; Karakawa et al., 2014). We recently found that CCR10 is critical for migration of T cells into the skin under steady-state conditions but is dispensable for their infiltration into inflamed skin (Fu et al., 2016; Xia et al., 2014). Like T cells, ILCs are also programmed in skin-draining lymph nodes to acquire the CCR10 expression for their migration to the skin under steady-state conditions (Yang et al., 2016). In CCR10-knockout mice, ILCs generated in skin-draining lymph nodes is defective in migration into the skin and increasingly diverted into spleens and other sites where CCR10 ligands are not expressed (Yang et al., 2016). Moreover, skin-specific CD207^+^ dendritic cells are involved in the programming of skin-homing CCR10^+^ ILCs in skin-draining lymph nodes (Yang et al., 2016) (Fig. 1).

Dependent on environmental cues of lymphoid organs in which T cells and ILCs are activated, they could be also imprinted to acquire homing properties to other distinct peripheral tissues (Campbell and Butcher, 2002; Kantele et al., 1999; Masopust and Schenkel, 2013; Mora et al., 2003; Mora et al., 2005; Mora and von Andrian, 2006; Rott et al., 1997; Weninger et al., 2000). A recent study reported that ILCs undergo a homing receptor switch by upregulation of CCR9 and α4β7 and downregulation of CCR7 in intestine-draining mesenteric lymph nodes in a retinoic acid-dependent manner for their migration into the intestine (Kim et al., 2015). Together with the findings in the skin, these results suggest that specific epithelial tissues and their associated lymph nodes form an integral unit for generation and maintenance of tissue-specific ILCs. However, there might be other regulatory mechanisms in establishment of tissue-specific ILCs (Kim et al., 2015; Yang et al., 2016). In our study of the generation of skin-specific CCR10^+^ ILCs in skin-draining lymph nodes, local inflammatory conditions suppress programming of skin-homing CCR10^+^ ILCs (Yang et al., 2016) (Fig. 1). Molecular factors regulating the distinctive generation and establishment of skin-specific ILCs under homeostatic and inflammatory conditions are mostly unknown.

## INTERACTION BETWEEN INNATE LYMPHOID CELLS AND LOCAL IMMUNE AND NON-IMMUNE CELLS IN PARTICIPATION OF SKIN INFLAMMATION

Compared to the healthy skin, ILC2s are increased in the skin of patients with atopic dermatitis (Kim et al., 2013; Salimi et al., 2013), suggesting that ILC2s are involved in atopic dermatitis, an inflammatory disease associated with over-active type 2 immune responses to environmental allergens and barrier dysfunction (Leung, 2013). In a mouse model of atopic dermatitis induced by topical application of Calcipotriene (a synthetic Vitamin D3 derivative), ILC2-derived IL-5 and IL-13 were reported to play an important role in promoting the disease development (Kim et al., 2013) (Fig. 1). It was also found that skin ILC2s are activated by TSLP produced by skin keratinocytes in response to the topical Calcipotriene-treatment (Kim et al., 2013). Another study identified the role of IL-33 and IL-25 in activating skin ILC2s to promote atopic dermatitis (Salimi et al., 2013). Consistent with this, transgenic overexpression of IL-33 in keratinocytes promotes accumulation of ILC2s in the skin and induces atopic dermatitis-like disease (Imai et al., 2013). Prostaglandin D2 (PGD2), acting through its receptor CRTH2 expressed on human skin ILC2s, induces migration and cytokine production of ILC2s in human atopic dermatitis skin (Xue et al., 2014). Mast cells could be an important source of prostaglandin D2 for activation of ILC2s (Barnig et al., 2013; Roediger et al., 2013). IL-4 derived from basophils was also shown to regulate the proliferation of skin ILC2s, which express the IL-4 receptor IL-4Rα, and induce atopic dermatitis-like disease (Kim et al., 2014a). Given the role of E-cadherin in inhibiting the cytokine production of ILC2s via binding to the inhibitory receptor KLRG1 expressed on skin ILC2s, downregulation of E-cadherin in atopic dermatitis skin might also contribute to the activation of skin ILC2s (Salimi et al., 2013).

Increasing evidence implicates involvement of ILC3s in psoriasis, a skin inflammatory disease largely resulting from overproduction of Th17-associated cytokines such as IL-17A (Dyring-Andersen et al., 2014; Pantelyushin et al., 2012; Teunissen et al., 2014; Villanova et al., 2014). In a mouse model of psoriasis induced by topical application of Imiquimod, it was found that ILC3s are a cellular source of IL-17A and IL-22 that mediate the Imiquimod-induced psoriasis-like disease (Pantelyushin et al., 2012). In patients with psoriasis, NKp44^+^ ILC3s are increased in both lesional and non-lesional skin and NKp44^−^ ILC3s have the potential to differentiate to NKp44^+^ ILC3s in response to stimulation of IL-1β and IL-23 (Teunissen et al., 2014; Villanova et al., 2014). Increased percentages of NKG2D^+^ RORγt^+^CD56^+^ ILC3s were found in the psoriatic skin in a separate study (Dyring-Andersen et al., 2014). There is a positive correlation between reduction of ILC3s and therapeutic response in psoriatic patients treated with anti-TNFα antibody (Villanova et al., 2014), supporting involvement of ILC3s in psoriasis. Cellular and molecular factors promoting activation of ILC3s in participation of psoriasis are still not well understood but could involve cytokines produced by myeloid, lymphoid, epithelial, and stromal cells of the skin (Gasteiger and Rudensky, 2014) (Fig. 1).

## CROSSTALK BETWEEN INNATE LYMPHOID CELLS AND OTHER SKIN IMMUNE CELLS IN MAINTENANCE OF LOCAL TISSUE HOMEOSTASIS AND HOST PROTECTION

While ILCs are relatively well studied for their participation in development of skin inflammatory diseases, their physiological role in maintenance of the skin homeostasis and host protection is still poorly understood. Since ILC2s are capable of producing amphiregulin, a member of epithelial growth factor family, they could potentially contribute to wound healing (Salimi et al., 2013). Similarly, IL-22 produced by ILC3s is an important growth factor of keratinocytes. Indeed, both ILC2s and ILC3s were recently reported to promote the wound healing process in mouse models (Li et al., 2016; Rak et al., 2016). The ILC2 response is promoted by the cutaneous injury in an IL-33-dependent manner (Rak et al., 2016), while ILC3s are recruited into wounded dermis by epidermal Notch1, which functions as a damage response signal (Li et al., 2016).

Skin ILCs interact with other skin-resident immune cells to maintain the immune homeostatic condition. In the steady-state skin, the preferential interaction of ILC2s and mast cells has been detected (Barnig et al., 2013; Roediger et al., 2013). IL-13 produced by skin ILC2s suppresses the function of mast cells in homeostatic conditions, suggesting a regulatory role of skin ILC2s (Roediger et al., 2013). ILCs might also interact with T cells to regulate their homeostasis in the skin (Yang et al., 2016), as reported in recent studies on the cross-regulation between ILCs and T cells in several other barrier tissues. ILC2s express MHCII and activate T cells to induce production of IL-2, which in turn promote the proliferation of ILC2s and their expression of type 2 cytokines for worm expulsion in the gut (Oliphant et al., 2014). In a model of allergic lung inflammation, IL-13 produced by ILC2s promotes the migration of dendritic cells to draining lymph nodes where they prime T cells to differentiate into Th2 cells (Halim et al., 2014). The co-stimulatory molecule OX40L and ILC2-derived IL-4 were reportedly involved in crosstalk between ILC2s and T cells (Drake et al., 2014). Intestinal MHCII-expressing ILC3s have the capacity to present antigens to CD4^+^ T cells and dampen their responses to microbiota (Hepworth et al., 2013). In addition, intestinal microbiota induces the production of GM-CSF by ILC3s, which promotes macrophages to produce retinoic acid and results in a tolerogenic state through enhancing function of regulatory T cells (Mortha et al., 2014). ILC3s could regulate the gut flora to control intestinal homeostasis and protect the host from intestinal pathogen infection (Goto et al., 2014; Guo et al., 2015; Qiu et al., 2013). Intestinal ILC3s also promote the immunoglobulin A antibody production by B cells through membrane lymphotoxin α1β2 (Kruglov et al., 2013). In our study of skin ILCs, we found that interaction between skin-specific CCR10^+^ ILCs and T cells plays a critical role in maintaining the immune homeostasis of the skin (Yang et al., 2016). In *Rag1* ^*−*/*−*^ mice that lack T and B cells, there are significantly reduced CCR10^+^ ILCs in the skin, while transfer of CD4^+^ T cells could partially restore the homeostatic presence of CCR10^+^ ILCs (Yang et al., 2016). Reciprocally, ILCs regulate homeostasis of resident T cells in the skin. When transferred into *Il2rg* ^*−*/*−*^ *Rag2* ^*−*/*−*^ mice, which lack all ILCs as well as T/B cells, donor CD4^+^ T cells in skin of hosts contain significantly higher percentages of IL-17A-producing Th17 and IL-5-producing Th2 cells but much lower percentages of IFNγ-producing Th1 cells than those transferred into *Rag1* ^*−*/*−*^ recipients that have ILCs (Yang et al., 2016). In absence of ILCs, there are also significantly reduced presence of regulatory T cells in the skin, indicating an important role of ILCs in regulating the homeostasis of skin T cells (Yang et al., 2016) (Fig. 1).

## PERSPECTIVES

The research over past several years has started to reveal roles of ILCs in the skin and unravel mechanisms regulating establishment and function of the skin-resident ILCs. However, several major questions remain to be addressed. First, physiological functions of skin-resident ILCs in host protection and immune homeostatic maintenance are still poorly understood. While ILCs have been generally implicated in protection against infection, control of commensal bacteria and regulation of immune activation in various barrier tissues, the function of resident ILCs specifically in the skin is not well studied. Considering that ILCs are maintained locally in different barrier tissues, functions and regulation of ILCs in the skin and other barrier tissues are likely regulated differently with local environmental cues. For example, the CCR10/CCL27 axis is involved in the regulation of resident ILCs in the skin but not other sites (Yang et al., 2016). Further studies are needed to understand how tissue-specific factors are involved in regulation and function of skin-specific ILCs. Associated with this, molecular mechanisms regulating tissue-specific establishment of ILCs in the skin versus other barrier tissues need further investigation. Furthermore, functions of different groups of ILCs in the skin require delineation at different ontogenic stages. For example, although group 1 ILCs, including conventional NK cells, are abundantly present in the skin of both humans and mice, particularly in the newborn and young (Batista et al., 2013; Ebert et al., 2006; Luci et al., 2009; Teunissen et al., 2014; Villanova et al., 2014), their roles in establishment of skin homeostasis are mostly unknown and need further investigation.

Second, mechanisms regulating differential involvement of ILCs in the skin homeostasis and inflammation are largely unknown. Studies of ILCs of the skin and other barrier tissues have showed that ILCs could be involved in both homeostatic regulation and tissue inflammatory processes. However, it is currently unknown whether and how ILCs involved in homeostatic regulation are different from ILCs involved in immune inflammatory processes in the skin. Our studies have found that CCR10^+^ ILCs are dominant in the healthy skin where they promote balanced maintenance of resident T cells (Yang et al., 2016). In immune dysregulatory or inflammatory conditions of Rag1^*−*/*−*^, Foxp3^*−*/*−*^ or topical Calcipotriol-treated wild-type mice, CCR10^+^ ILCs are decreased while CCR10^−^ ILCs are increased in the skin (Yang et al., 2016), suggesting that CCR10^+^ and CCR10^−^ ILCs might be differently involved in skin immune homeostatic regulation and activation (Fig. 1). Further study on how CCR10^+^ and CCR10^−^ skin ILCs are regulated and function should help understand basic biology of the skin-specific ILCs in the health and disease.

Third, the involvement of ILCs in promoting diseases has raised interest in modulating ILC functions for the treatment of diseases. In other tissues, antibodies against ILC2-activating cytokines, such as IL-25 and IL-33, as well as those targeting ILC2-produced cytokines, including IL-5 and IL-13, have been found to suppress ILC2 proliferation and function in tissue inflammatory diseases in the lung (Gauvreau et al., 2014; Hambly and Nair, 2014). Blockage of the binding of prostaglandin D2 to CRTH2 expressed on human ILC2s inhibits the migration and cytokine production of ILC2s (Barnig et al., 2013; Xue et al., 2014). Montelukast, a leukotriene receptor antagonist, inhibits the cytokine production of ILC2s by blocking interaction between leukotriene D4 and cysteinyl leukotriene receptor 1 (CysLT1R) expressed by ILC2s (Doherty et al., 2013). ILC3s have been targeted in the treatment of multiple sclerosis by anti-IL-2R antibody (Perry et al., 2012). Antagonists of RORγt, which can regulate the activity of Th17, might also target ILC3s (Bernink et al., 2015; Solt et al., 2011; Withers et al., 2016). However, while there is a report showing that inhibition of RORγt reduces differentiation of CD127^+^ ILC1s to ILC3s (Bernink et al., 2015), inhibition of RORγt does not impair function of mature ILC3s (Bernink et al., 2015; Withers et al., 2016). How to target ILCs in the skin to modulate their cytokine production for treatment of skin inflammatory diseases is still understudied and might represent a new area of study for disease treatment.

## ABBREVIATIONS

αLP: α-lymphoid precursor
CHILP: common helper-like innate lymphoid progenitor
CILP: common innate lymphoid progenitor
EILP: early ILC progenitor
Id2: inhibitor of DNA binding 2
IFNγ: interferon γ
IL-2: interleukin-2
ILCP: innate lymphoid progenitor
ILCs: innate lymphoid cells
LNs: lymph nodes
LTi: lymphoid tissue inducer
MHCI: class I major histocompatibility complex
NCR: natural cytotoxicity receptor
NK: natural killer
PGD2: prostaglandin D2
PLZF: promyeloid leukemia zinc finger
SCID: severe combined immunodeficiency
Th: helper T
TNFα: tumor necrosis factor α
TSLP: thymic stromal lymphopoietin
